# Evaluation of Human Resources for Nursing Care in Bhutan: A Situational Review

**DOI:** 10.1002/puh2.70090

**Published:** 2025-08-01

**Authors:** Sangay Tenzin, Arun Gautam, Julendra Koriala, Tenzin Choden, Krishna Maya Poudel, Amber Bahadur Gurung, Monu Tamang, Thinley Dorji, Dorji Gyeltshen, Nidup Dorji

**Affiliations:** ^1^ Department of Nursing Central Regional Referral Hospital Gelephu Bhutan; ^2^ Department of Physiotherapy Central Regional Referral Hospital Gelephu Bhutan; ^3^ Graduate School of Public Health International University of Health and Welfare Narita Japan; ^4^ Department of Internal Medicine Central Regional Referral Hospital Gelephu Bhutan; ^5^ Apollo Bhutan Institute of Nursing Thimphu Bhutan; ^6^ Faculty of Nursing and Public Health Khesar Gyalpo University of Medical Sciences of Bhutan Thimphu Bhutan

**Keywords:** attrition, health services, human resources, nursing education, psychological burnout, workforce

## Abstract

Adequate nursing workforce is essential to ensure the delivery of effective and quality nursing care. However, the shortage of nurses accounts for more than half of the total shortage of healthcare professionals worldwide. Bhutan is one such country that reported an alarming 9.14% attrition of nursing workforce in 2022–2023. The average density of nurses in Bhutan has declined from 21.07 per 10,000 population in 2021 to 20.42 nurses by March 2023. Prior to the current high rate of attrition of nurses, the Ministry of Health had projected the need of additional 1595 nurses by 2026 to meet the minimum desired nurse to population ratio. With an increasing number of nurses leaving for developed countries seeking better economic opportunities, the numbers of nurses are expected to decline in Bhutan. As of 2025, nursing education in Bhutan is provided through one government and three private training colleges. Despite this, it is unlikely that the gap in nursing workforce requirement will be met as graduates prefer to migrate to developed countries. Some of the factors leading to attrition are poor remuneration, lack of clear job descriptions and lack of job satisfaction. Nurses who lack bachelor's degree face difficulties in career progression and advancement. We recommend the development of clear job description, introduction of nursing licensing examination for regulatory oversight and diversification of the roles of nurses into school and community‐based programmes. With the reforms in the health system, health policymakers and health administrators need to develop a long‐term plan for sustainable health human resource development. This Perspective presents a brief history of nursing profession, current situation of the nursing human resource, attrition and its contributing factors and the implications for Bhutan.

## Graphical abstract

This figure provides a visual summary of the changes in Bhutan's active nursing workforce between January 2022 and January 2023. In January 2022, the workforce stood at 1446 nurses. By January 2023, this number had increased to 1485, reflecting a net gain of 39 nurses over the oneyear period. This growth occurred despite notable workforce attrition: 81 nurses resigned, and 53 went on extraordinary leave, totalling 134 exits. On the basis of this, it can be inferred that approximately 173 new nurses were recruited during the year to not only replace those who left but also to achieve the net workforce increase. However, this growth in the nursing workforce remains insufficient to meet the intended target number of nurses required. The data suggest an attrition rate of around 9.14%, which, while not critical, points to a moderate level of instability that could impact service delivery if not addressed. The figure underscores the importance of not only continuing recruitment efforts but also strengthening retention strategies to reduce the number of resignations and manage long‐term absences. It highlights the need for effective workforce planning, policy interventions and support mechanisms to ensure a resilient and sustainable nursing workforce in Bhutan.

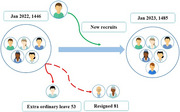

## 1 Introduction

A health workforce with sufficient capacity, capability and quality to meet the challenges and demands in the community and in healthcare settings is critical for universal health coverage [1]. Globally, there are 27 million nurses and midwives, making them the largest healthcare professional groups in most countries and comprising half the global healthcare workforce [1]. In recent years, there was a significant progress in South East Asia region in the recruitment and the training of nurses from 2.9 million, 16 per 10,000 population in 2014 to 3.5 million, 18 per 10,000 population in 2018 [2]. However, the shortage of nurses also contributes to more than 50% of total global shortage of health workforce with the largest shortage in South East Asia and Africa [3]. The number of nurses in South East Asia region is far below the global average of 37 nurses per 10,000 population and requires an addition of 1.9 million nurses by 2030 [2]. This shortage is exacerbated by nurses leaving the job in their country for better economic opportunities offered elsewhere. Popular destinations for Indian and Nepalese healthcare professionals are the Gulf Cooperation Council (GCC) countries, Australia, the United States and Singapore [4].

Bhutan—a lower middle‐income country in South East Asia—has a long‐standing problem with shortage of nursing workforce exacerbated by nurses with work experience leaving for Australia, Canada and Singapore. This Perspective reviews nursing human resources and factors perpetuating the chronic shortage of nurses in Bhutan.

### 1.1 Development of Nursing Services in Bhutan

After establishing modern healthcare system in the country in early 1960s, nurses evolved as a major healthcare workforce [5]. In the beginning, a few village girls were provided on‐the‐job training by the expatriate nurses to perform basic care and housekeeping in hospitals as well as engaged them in comprehensive health promotion as part of the Primary Health Care programme [6].

The Health School was established in Thimphu in 1974 as a centre for training of health human resources, with assistance from the United Nations Children's Fund (UNICEF) and the World Health Organization. The first batch of pre‐service certificate programme of Auxiliary Nurse and Midwifery (ANM) was started in 1975 with the objective of posting them in Primary Health Centres. In 1978, another certificate programme for Assistant Nurse (AN) was introduced and was phased out in 2000 [6]. The ANM programme was merged with the Health Assistant (HA) programme in 2001.

In 1982, the 3.5 year diploma programme in General Nursing and Midwifery (GNM) was started with a batch of 10 students [5]. In 2001, the Faculty of Nursing and Public Health (FNPH) of Khesar Gyalpo University of Medical Sciences of Bhutan (KGUMSB) started offering a two year conversion course for GNM to obtain bachelor's degree in collaboration with La Trobe University, Australia. The first batch of nurses pursuing conversion course for Bachelors of Nursing graduated in 2003 [6]. Today the programme is independently delivered by the KGUMSB. The university also offers 6‐month specialization courses in adult, paediatric and neonatal intensive care nursing, dialysis nursing and peri‐operative nursing for in‐service candidates.

In 2014, to address the shortage of nurses in Bhutan, the government has approved private nursing colleges. The Diploma in Nursing and Midwifery has been initiated at the Arura Academy of Health Sciences in 2014 and at the Apollo Bhutan Institute of Nursing in 2019. Bachelor degree in nursing and midwifery has been initiated at the Royal Thimphu College in 2018. Additionally, nurses undergo bachelor's and master's degrees in nursing in India and Thailand. Lately, Bhutanese nurses also pursue their higher degrees in Australia and Japan.

### 1.2 Nursing Workforce in Bhutan

**FIGURE 1:**
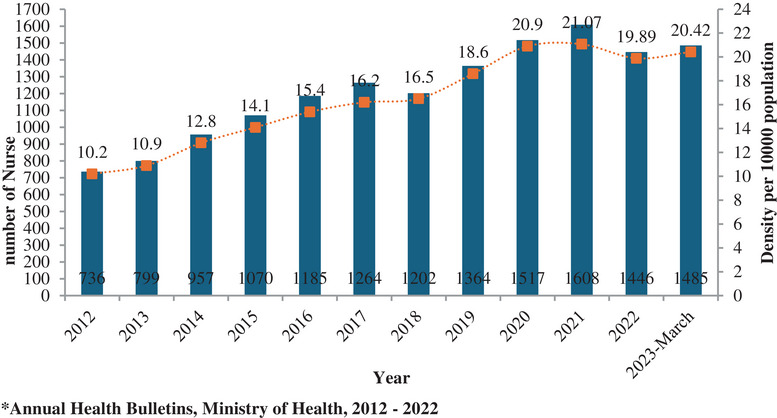
The trend of nursing human resource and density of nurses per 10,000 population in Bhutan, 2012–2023.

Nurses are the largest group of healthcare workforce, and the country has seen an average annual growth rate of 1.26% in the last 10 years (Figure 1) [6]. In 2022, Bhutan had 1608 registered nurses and midwives, with an average of 21.07 nurses per 10,000 population [7], which is significantly low in comparison to developed countries like Finland and Sweden with more than 200 nurses per 10,000 population [8]. However, the number of nurses decreased to 1485 in March 2023 reflecting a significant loss of workforce after the COVID‐19 pandemic.

Bhutan has a three‐tiered state‐sponsored healthcare system [7]. As per the national health human resource deployment standards, nurses are deployed up to the primary level healthcare centres. Thimphu, Sarpang and Mongar districts record a higher number of nurses where the referral hospitals are located (Table 1).

**TABLE 1:** The number of nurses available across the 20 districts in Bhutan, March, 2023.

Dzongkhag	Population^a^	Number of nurses	Nurses per 10,000 population
Bumthang	17,820	13	7.29
Chhukha	68,966	99	14.35
Dagana	24,965	31	12.42
Gasa	3952	4	10.12
Haa	13,655	11	8.05
Lhuentse	14,437	20	13.85
Monggar	37,150	147	39.57
Paro	46,316	30	6.48
Pema Gatshel	23,632	27	11.43
Punakha	28,740	26	9.05
Samdrup Jongkhar	35,079	60	17.1
Samtse	62,590	82	13.1
Sarpang	46,004	163	35.43
Thimphu	138,736	530	38.2
Trashigang	45,518	68	14.95
Trashi Yangtse	17,300	21	12.14
Trongsa	19,960	34	17.03
Tsirang	22,376	36	16.1
Wangdue Phodrang	42,186	54	12.8
Zhemgang	17,763	33	18.58
Total	**727,145**	**1485**	**20.42**

^a^ Population and Housing Census of Bhutan 2017, National Statistics Bureau, Bhutan.

Due to the lack of separate cadre for nurses and midwives in the Royal Civil Service Commission, both nurses and midwives are referred to as ‘nurses’ in Bhutan [5]. The Royal Civil Service Commission, the sole employer of nurses in the country, categorises nurses into three levels according to their qualification: Those with at least bachelor's degree are called Clinical Nurses, those with diploma‐level qualification are called Staff Nurses and those with certificate‐level qualification are called Assistant Nurses. Despite differences in qualifications, due to ambiguity in the description of roles and scope of practice in patient care settings, all categories of nurses perform almost the same level of tasks at the workplace [9]. Today, nurses also occupy central roles in infection control, quality assurance, regulatory bodies and teaching nursing in the university and colleges [5, 9].

### 1.3 Shortage, Attrition and Its Implications

**FIGURE 2:**
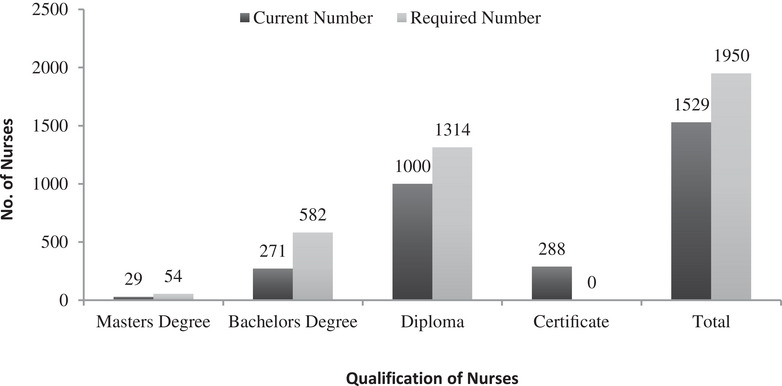
The gap in the nursing and midwifery workforce in Bhutan. *Source:* National Strategic Direction for Nursing and Midwifery 2021–2025, MoH 2021.

The unprecedented shortage of nurses is a global issue resulting from the migration of nursing workforce from the low‐ and middle‐income countries to high‐income countries [10], resulting in an inequitable distribution of the nurses globally [11]. The situation is no different in Bhutan with the shortage of more than 600 nurses [5] with a projected requirement of 1595 additional nurses by 2026 to meet the minimum desired nurse to population ratio (Figure 2) [12].

**FIGURE 3:**
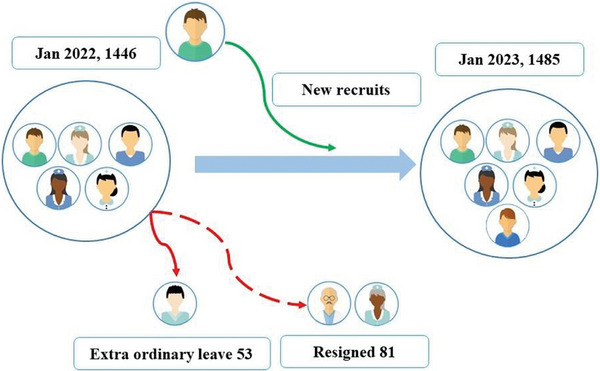
Comparison of active nursing work force in Bhutan between January 2022 and January 2023.

The shortage of nurses in Bhutan has been aggravated by the increasing attrition rate, with highest attrition reported in 2018–2019. Nurses leaving the job were negligible in 2020, which could be attributed to the travel ban during the COVID‐19 pandemic. After lifting the travel restrictions, Bhutan has been witnessing an exponential rise in the number of nurses leaving the job. In the first month of 2023, a total of 15 nurses have resigned within 11 days [13]. The Jigme Dorji Wangchuck (JDW) National Referral Hospital alone has seen the highest attrition of more than 50 nurses in just the first half of 2022 [14]. Between 2019 and 2022, 374 nurses left their job among whom 323 nurses resigned voluntarily to seek better opportunities in developed countries, especially Australia [13]. The attrition rate in March 2023 stood at 9.14% compared to its annual growth rate of 2.6% (Figure 3).

As of May 2024, the national level rate of attrition stands at 25% posing intense pressure on the healthcare system of the country. The JDW National Referral Hospital is facing an acute shortage of nurses with 70 nurses performing job which was previously done by 100 nurses, with attrition nearing 30% [15]. The shortage of nurses has detrimental implications on quality of patient care and healthcare expenditure [10]. In developing countries, nursing shortages have resulted in increased workload, delays in patient care, high turnover rates, incorrect job shifting, subpar patient outcomes, occupational injuries and malpractices [16]. The shortage of nurses is directly proportional to burnout and emotional exhaustions among the nurses [17]. The emotional exhaustion and burnout compromises provision of quality nursing care to the patients [18] or omission of patient care [19] leading to longer hospital stay [20] and higher patient mortality [18]. Furthermore, the risk of treatment errors and adverse events during the treatment process is higher in understaffed units compared to the units with adequate nurses [21]. On the other hand, higher nurse‐to‐patient ratio has been shown to improve the quality of patient care leading to lower morbidity and mortality [21, 22].

The quality of the patient care at the JDW National Referral Hospital is affected with the current nurse–patient ratio of 1:16 compared to the 1:6 earlier, directly related to the increasing attrition rate of nurses [22]. Nurses work a 12‐hour shift instead of six‐hour with their leave provisions cancelled due to the shortage of staff [13], compromising quality of the patient care and increasing errors and patient dissatisfaction as evidenced by increasing patient complaints in media and social media. A study done at the JDW National Referral Hospital reported that the medication errors are quite high, prompting urgent administrative actions to address it [23].

### 1.4 Factors Leading to Nurse Attrition

The labour market influences nurses’ choices about their employment. Many travel and work in other countries for better economic and career opportunities and better working conditions [24]. News articles in Bhutan report low pay, burnouts and the fear of professional errors due to overwork [25] and staff shortage as some of the common reasons for departure from work [13]. The lack of clear job descriptions and lack of job satisfaction were some of the other reasons [26]. English language proficiency among Bhutanese nurses makes it easier for them to migrate and work in the English‐speaking countries [4]. English is the medium of instruction in Bhutan as well as other countries such as India and Thailand where most of the Bhutanese nurses undergo trainings.

Attrition of nurses is not a linear problem but a multi‐faceted phenomenon. As such, comprehensive research on attrition of nurses is urgently warranted to develop and implement pragmatic and evidence‐based strategies to retain nurses in the country.

### 1.5 Addressing the Shortage of Nurses

The National Medical Services, the nodal agency responsible for providing clinical services in the country, has extended the resignation notice period for the clinical staff to 6 months to prevent a potential collapse of the health services [27]. The government plans to hire foreign nurses to address the acute shortage of nurse in JDW National Referral Hospital which will cost $1000 per month for a nurse [28]. However, there are concerns of pay disparity between the Bhutanese nurses and the expatriates for the same job [29], where Bhutanese nurses are paid $470 per month [28]. Additionally, language barriers with expatriate nurses would hamper effective communication resulting in compromised quality of care, patient dissatisfaction and requirement of translators [30]. Such disparities might result in further attrition of nurses from the healthcare system.

In the workplace, there are ambiguities in the role and scope of practice, deployment and remuneration based on qualifications and experiences [5]. The lack of appropriate remunerations and recognition has led to a lesser number of nurses to upgrade their qualification. For example, ANs have remained stagnant in their career with no opportunities of upgrading their qualification, leading them to pursue public health or administration for advancement of their education despite their interests in the field of nursing. Though Nurse Anaesthetists play a critical role in the background of shortage of anaesthesiologists, they also lack opportunities to upgrade their qualification. It is crucial to design education and career development pathways within the civil service to retain the skilled nurses.

In Bhutan, the nurses are regulated by the Medical and Health Professionals Council, Bhutan Qualifications and Professional Certification Authority. Nursing regulation and strategic planning of nursing development require specific focus and dedicated resources. We recommended that a separate section for nursing services may be required at the regulatory office to bring about professional development and improve nursing standards at par with international standards. The development and implementation of a national licensure examination for nurses and midwives could be viewed as a tool to raise educational standards [31].

Despite the nurses and midwives being trained with a diverse scope of practice to provide essential healthcare services at all levels of health facilities, the current deployment of the nurses in Bhutan is limited to the hospitals [5]. Such constraints in the scope of practice also result in reduced job satisfaction. For example, school health nurse or an occupational health nurse has major scope of bringing benefits to the wider communities [32]. To meet these requirements, there is a need to review the nursing curriculum to reorient the roles of nurses in the primary health centres [9]. Additionally, nurses also play an important role in empirical and clinical research.

## 2 Conclusion

Bhutan faces major challenges with an acute shortage of nurses in the background of high attrition rates demanding urgent attention. Increased workload, lack of clear job distinctions based on qualifications and inadequate remuneration are some of the key factors contributing to attrition. Without immediate action, these issues will only worsen, leading to further strain on the healthcare system and diminishing the quality of patient care.

To address the shortage of nurses, there is a need for revision of human resource planning to ensure that recruitment, training and retention strategies are aligned with the country's needs. Additionally, providing adequate incentives and providing opportunities for nurses to have a meaningful engagement in their career are critical in reducing and fostering a motivated workforce. The consequences of inaction are clear: Continued shortages and a lack of support for nurses could result in burnout, a decline in quality of care and a negative impact on Bhutan's overall health indicators. It is imperative that policy interventions are implemented to ensure a sustainable, effective and resilient nursing workforce in Bhutan.

## Author Contributions

All authors were involved in the conception of this topic. Sangay Tenzin, Monu Tamang, Nidup Dorji and Thinley Dorji wrote the manuscript. Arun Gautam collected the data and designed the figure and tables. All authors were involved in critically reviewing and editing the manuscript. All authors have approved the manuscript for publication.

## Ethics Statement

The authors have nothing to report.

## Consent

The authors have nothing to report.

## Conflicts of Interest

T.D. is the editorial member of this journal. He was blinded from peer review process at all stages.

## Data Availability

Data sharing is not applicable to this article as no datasets were generated or analysed during the current study.
